# An Overview of Biological Macromolecule Crystallization

**DOI:** 10.3390/ijms140611643

**Published:** 2013-05-31

**Authors:** Irene Russo Krauss, Antonello Merlino, Alessandro Vergara, Filomena Sica

**Affiliations:** 1 Department of Chemical Sciences, University of Naples Federico II, Complesso Universitario di Monte Sant’Angelo, Via Cintia, Napoli I-80126, Italy; E-Mails: irene.russokrauss@unina.it (I.R.K.); antonello.merlino@unina.it (A.M.); alessandro.vergara@unina.it (A.V.); 2 Institute of Biostructures and Bioimages, C.N.R, Via Mezzocannone 16, Napoli I-80134, Italy

**Keywords:** protein, nucleic acid, membrane, crystallization, new methodologies

## Abstract

The elucidation of the three dimensional structure of biological macromolecules has provided an important contribution to our current understanding of many basic mechanisms involved in life processes. This enormous impact largely results from the ability of X-ray crystallography to provide accurate structural details at atomic resolution that are a prerequisite for a deeper insight on the way in which bio-macromolecules interact with each other to build up supramolecular nano-machines capable of performing specialized biological functions. With the advent of high-energy synchrotron sources and the development of sophisticated software to solve X-ray and neutron crystal structures of large molecules, the crystallization step has become even more the bottleneck of a successful structure determination. This review introduces the general aspects of protein crystallization, summarizes conventional and innovative crystallization methods and focuses on the new strategies utilized to improve the success rate of experiments and increase crystal diffraction quality.

## 1. Introduction

Genome-sequencing projects have provided a near complete list of the molecules that are present or potentially present in an organism, and post-genomic projects are aimed at cataloguing the relationships between them. Understanding metabolic and signaling pathways or gene-regulatory networks relies on a detailed knowledge of protein-metabolite, protein-protein and protein-nucleic acid interactions. X-ray diffraction from high quality crystals remains the most reliable approach to obtain detailed structural information that provides powerful insight into the molecular mechanisms underlying the function of bio-macromolecules and the way they interact to form complex supramolecular assemblies [1]. Furthermore, knowledge of binding sites at atomic details allows a rational drug design, which is fundamental for searching of new medicines [2]. For this purpose, protein crystallography is now used at all levels, including target identification and selection [3–5].

The comparison between the number of non-redundant protein sequences deposited in Uniprot (>20 million) and protein structures deposited in the PDB (about 90,000, as of April 2013) clearly shows that the challenge in determining the crystal structure of all the proteins from an organism is arduous. The determination of the 3D structure by X-ray crystallography involves essentially six steps: (I) purification from source or cloning, expression and purification of target macromolecule; (II) search of initial crystallization conditions; (III) optimization of crystal quality; (IV) diffraction data collection; (V) structure determination and refinement of the 3D model; (VI) analysis of the refined model. The advent of high-throughput methods has made the process more efficient [6,7]. Indeed, notable advances in tools for X-ray data collection have been made, including synchrotron beam lines [8], sensitive X-ray detectors, and improved cryogenic and mounting procedures for crystals. Once good quality crystals are obtained, the subsequent steps of structure determination can be more safely carried out. However, many times the high-throughput protein structure determination does not give positive results, due to the difficulties of finding conditions that promote growth of high-quality protein crystals.

**Figure 1:**
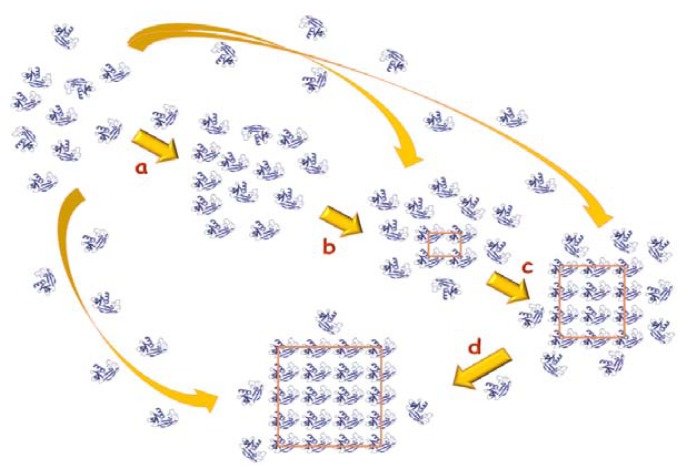
Fluid aggregate model. Increasing supersaturation promotes molecule association, which begins to organize into large disordered aggregates (**a**) molecules within the cores of such aggregates reorient, redistribute and form more geometrically rigorous interactions; (**b**) These latter interactions tend to order and stabilize the aggregate core, which increases to produce a critical nucleus; (**c**) This ultimately develops into a true crystal; (**d**) Free molecules are then adsorbed to the crystal surface and increase its size by their incorporation into its lattice.

The three stages of crystallization common to all molecules (Figure 1) are nucleation, crystal growth and cessation of growth [9–12]. During nucleation an adequate amount of molecules associate in three dimensions to form a thermodynamically stable aggregate, the so called critical nucleus, which provides surfaces suitable for crystal growth. The growth stage, which immediately follows the nucleation, is governed by the diffusion of particles to the surface of the critical nuclei and their ordered assembling onto the growing crystal (Figure 1).

Protein crystal formation requires interactions that are specific, highly directional and organized in a manner that is appropriate for three-dimensional crystal lattice formation. Crystal growth ends when the solution is sufficiently depleted of protein molecules, deformation-induced strain destabilizes the lattice, or the growing crystal faces become poisoned by impurities. The crystallizability of a protein is strictly affected by the chemical and conformational purity and the oligomeric homogeneity of the sample.

**Figure 2:**
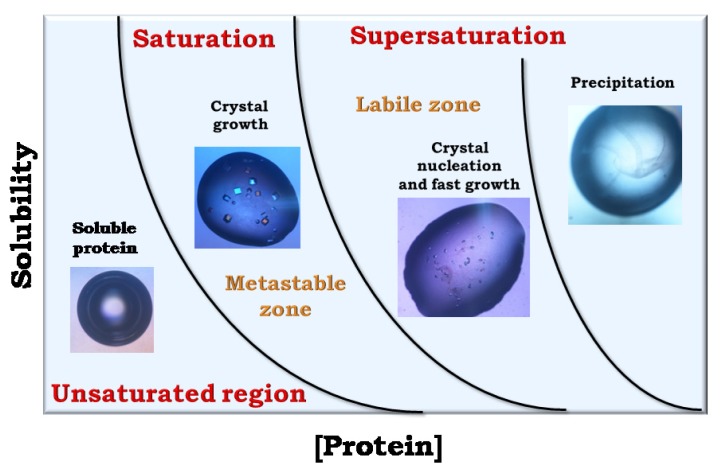
Schematic solubility curve for a protein, as a function of the protein concentration.

The whole crystal growth process can be conveniently visualized in a two-dimensional phase diagram (Figure 2) representing the stable states (liquid, crystalline, precipitate) as a function of two crystallization variables. When the concentration of a protein solution is brought above its solubility limit, the solution becomes supersaturated. Depending on the level of supersaturation this zone of the diagram can be divided into three regions: very high supersaturation (“precipitation”), where molecules form amorphous aggregates [13], intermediate supersaturation (“labile”), where both growth and nucleation occur, and lower supersaturation (“metastable”), where only growth is supported. Because these regions are related to kinetic parameters, the boundaries between them are not well defined.

The best strategy that should be employed is to induce nucleation at the lowest level of supersaturation just within the labile region. Following nuclei formation, the concentration of protein in the solution gradually decreases, driving the system into the metastable zone, where growth occurs slowly. However, it is very difficult to identify these ideal conditions and in order to obtain high-quality crystals it could be necessary to physically separate nucleation and growth steps. Chemical space in crystallization experiments is multidimensional, and several zones may correspond to nucleation and growth of different crystal forms. It is not yet possible to predict the conditions required to crystallize a protein from its chemico-physical properties. Changes in a single experimental parameter can simultaneously influence several aspects of a crystallization experiment.

Despite the importance of protein crystallization, the insights into this process are still limited and currently there are no systematic methods to ensure that ordered three-dimensional crystals will be obtained. This problem has stimulated many efforts to improve the success of protein crystal growth experiments.

The present work will review some of the basic ideas and principles of biological macromolecule crystallization, summarize the standard approaches in crystal growth and illustrate novel tools and strategies to increase the rate of positive results and the diffraction quality of crystals.

This manuscript focuses mainly on soluble proteins, but covers also the crystallization of membrane proteins, nucleic acids and nucleic acids/protein complexes, and discusses general physico-chemical aspects of the crystallization mechanisms.

## 2. Factors Affecting Crystallization of Bio-Macromolecules

Crystal growth of bio-macromolecules is a multi-parametric process as it depends on several factors, such as sample concentration, temperature, pH value, precipitant, buffer, additive, detergent, physical fields, pressure *etc*. (Table 1).

All these thermodynamic and dynamics variables can be used to control supersaturation in the system and, thus, indirectly, the rates of nucleation and growth. In the following sections we will describe in details some parameters affecting protein crystallization, which also govern crystallization of other biological macromolecules, such as nucleic acids or macromolecular assemblies.

### 2.1. Sample Purity and Homogeneity

Chemical and conformational purity of the sample strongly affects the ability to grow crystals. It can be simply assessed by sodium dodecylsulfate-polyacrylamide gel electrophoresis and mass spectrometry (MS). As regards the secondary structure analysis, two techniques are currently used: Fourier Transform infrared Spectroscopy (FT-IR) and UV circular dichroism (UV-CD). However, UV-CD is the most useful, mainly due to the high concentration of sample required for FT-IR studies [16]. Sample homogeneity, another fundamental precept of crystallization, can be determined with dynamic (DLS) and/or static light (SLS) scattering. The light scattered from a solution may be analyzed either in terms of its intensity or in terms of its fluctuations. In the former method, which is called static light scattering, the measure of scattered light intensity as function of angle is used to find the molar mass, the mean squared radius of gyration and the second viral coefficient (B_22_). The measurements may be also performed at a single angle, provided that the concentration and the refractive index are known. A B_22_ value in the range −0.8 × 10^−4^ to −8 × 10^−4^ mol mL g^−2^ indicates the possibility of a successful crystallization, whereas a value outside this range will probably result in crystallization failure. This procedure has been used to investigate solubility and crystallizability of many macromolecules [17–20]. The results of SLS experiments can be used as a quality control of protein preparation and to investigate the solution oligomeric state (monomer/dimer, *etc.*). On the other hand, DLS detects the fluctuations of the scattering intensity due to the Brownian motion of molecules in solution [21,22]. The degree of these fluctuations depends on the diffusion coefficient of the scattering particle, a quantity which is related to the hydrodynamic radius through the Stokes-Einstein equation. The mathematical treatment of the data also permits to assess the degree of homogeneity (polydispersity index) as well as to construct size distribution. Furthermore, Saridakis *et al.* [23] demonstrated that the DLS-based separation of nucleation and crystal growth processes can result in protein crystals with improved size.

### 2.2. Temperature

Temperature governs the balance between enthalpy and entropy effects on the free energy. Depending on whether crystallization is enthalpy- or entropy-driven, proteins may become either more or less soluble at higher temperature. Some proteins display a characteristic increased solubility with increasing temperature, whereas others display a decreased, or retrograde solubility [24]. The dependence of protein solubility on temperature is due to the variation of the acid/base reaction constant of the protein side chains as function of temperature [25]. Furthermore, pKa values of ionizable groups are strictly related to the medium ionic strength. As a consequence, in the case of proteins with normal solubility, it increases with a temperature increment at low ionic strength, for example if the solution contains components with low dielectric constant, whereas decreases at high ionic strength. In the latter case, however, the solubility variation is very small. The temperature-solubility function is not a property of the protein itself, but is subtly related to the protein-solution system. Equally relevant is the influence of temperature on the rates of nucleation and growth, and on the equilibrium position of the trial. Generally, in a crystallization laboratory, experiments are performed at two different temperatures (4 °C and 20 °C). However, recently many crystallization devices with a fine control of the temperature have been developed to take advantage of the effects of this parameter on the growth mechanism and the crystal form.

### 2.3. pH

Proteins generally contain numerous ionizable groups, which have a variety of pKas. As a consequence, protein solubility can dramatically change as pH is altered even by only 0.5 pH units and in some cases it varies for very small pH changes (0.1 units). The pH affects the detailed nature of protein-protein interactions modifying the possibilities of forming salt bridges and hydrogen bonds crucial to the formation of specific crystal contacts [26]. Electrostatic interactions, which depend on the protonation state of aminoacid side chains, play a key role in the binding specificity, in protein hydration and in the interactions with small molecules and ions that sometimes mediate the crystal packing contacts. At a pH characteristic for each protein, called the isoelectric point (pI), the positive charges of the molecule exactly balance the negative ones. This would seem to be the best situation for crystal growth as no overall electrostatic repulsion between protein molecules is present. Unfortunately, this idea was not confirmed by an analysis of crystallization conditions of almost ten thousand unique protein crystal forms [27]. Consequently, a wide pH range has to be explored in the crystallization experiments, but only pH values that maintain the folded structure of the protein are acceptable conditions for protein crystal growth.

### 2.4. Thermal Stability

The correlation between protein thermal stability and probability of yielding crystals is controversial [28]. However, in many cases pre-crystallization screening based on stability has substantially increased the crystallization success rate [29,30]. A rapid and low-cost method able to determine protein stability is the fluorescence-based thermal shift assay, also referred to as a “differential scanning fluorimetry” (DSF). This method measures the melting temperature of a protein by monitoring the signal of an external fluorescent probe which interacts with hydrophobic core residues when they become solvent-exposed during the unfolding process [31]. The low quantity of starting material required for an average thermal shift experiment makes DSF particularly suitable for use in the screening of optimal conditions for protein crystallization targets.

### 2.5. Precipitant

Chemical compounds that reduce protein solubility are referred to as crystallizing (or precipitating) agents. They reinforce the attractions among bio-macromolecules and act either by altering the activity coefficient of water (salts) [32], or by changing the dielectric constant of the solvating medium (organic solvents) or by increasing molecular crowding (high molecular weight polymers like polyethylene glycol, PEG) [33]. Precipitants that act by different mechanisms show little exchangeability: crystals obtained with one type of precipitant do not commonly form if the precipitant is changed with a functionally different one. However, it has been exhaustively demonstrated that combinations of mechanistically distinct precipitating agents can be synergistic and increase the probability of crystal growth [34] (Table 2).

### 2.6. Additives

A common approach in crystallization strategies is the use of additives to affect crystal growth and nucleation. Any substance other than the crystallizing compound, the buffer and the precipitant agent is considered as an additive (Table 3).

An additive can bind, modify and/or stabilize the protein conformation, perturb protein-protein and protein-solvent interactions, influence the various crystallization phases and change surface energy of the crystal [14,36]. The most common interpretation of specific effects of a given additive on crystal growth is based on the assumption that some specific interactions are established between the additive and particular sites at the protein surface. Very frequently, small salts are used as additive in the crystallization solution. For most proteins the degree of solubility depends weakly on the kind of cation, but strongly on the kind of anion [37]. In particular, the so-called “Hofmeister series” sets a well-defined empirical scale of efficiency for the different ions in precipitating proteins from solution [32,38]. In some cases, the ions are essential for the protein biological activity and contribute to maintain certain structural features of the protein. In other cases, metal ions stabilize intermolecular contacts in the crystal. Recent studies have shown that the application of biocompatible water-soluble ionic liquids, organic salts and salts with melting points at or below room temperature as crystallization additives provides very interesting results [39,40]. Small organic molecules represent another class of very useful additives, which increase long range electrostatic interactions by lowering the dielectric constant, affect solvent structure, and could modify the hydrophobic effect [41–45]. Crystallization of a certain protein often requires the presence in the crystallizing solution of natural ligands, which assure the conformational homogeneity of the sample. These ligands can be chosen on the basis of their binding affinity to the enzyme. Some of these compounds can be added directly to the crystallization drop (co-crystallization), others need to be bound to the protein in advance. Since the pioneering studies on additive effects on protein crystal growth [36,46,47], the use of these molecules has become extremely widespread. In the literature many papers discuss the action and the effects of these substances on the protein crystallizability and very recently McPherson *et al.* have suggested that these small reagents, and their combinations, may play a primary role in the crystallization process [35].

A new procedure for the use of additives has been recently proposed that is applicable to standard vapor diffusion sitting and/or hanging drop methods [48,49]. In this technique, called the cross-influence procedure (CIP), each crystallization trial utilizes four droplets containing equal volume of the precipitating agent. The protein is added to one of the droplets whereas additives (metal salts) are placed in the others, then all drops are left to equilibrate against the same reservoir. The presence of the drops without sample should slightly change the vapor pressure of water over the drop containing the protein. Furthermore, when the precipitant solution contains volatile buffers, the pH of the drop with the protein may slightly change, due to vapor diffusion of the volatile acid or base component. On the basis of the results so far obtained, this method appears to be very promising and can become highly effective for finding and/or improving crystallization conditions.

### 2.7. Gravity

During the crystal growth, the solution around the crystal gets depleted in protein and becomes less dense than the bulk solution. Therefore a density gradient is created that in conjunction with the gravitational field leads to buoyancy driven convection close to the crystal [50]. Furthermore, the growing crystal surfaces come in contact with bulk solution that is typically several times supersaturated. These effects are harmful, because they interfere with the correct addition of protein molecules to the growing crystal lattice and may cause crystal disorder. In zero gravity no buoyancy-driven convection occurs and the growing crystal does not move with respect to the surrounding fluid. The matter is transported in a purely diffusive way [51] and the crystal growth takes place under ideal conditions where the growing surface is in contact with a solution that is slightly supersaturated. Another negative effect of gravity on growing crystal is sedimentation that may induce the partially formed crystals to fall on top of one another where they continue to grow. However, the results of crystallization experiments performed in space are controversial and indicated that about 30% of space-grown protein crystals yield better X-ray diffraction data than the best crystals grown on the earth [50,52,53]. This because aboard orbiting space crafts all objects experience forces that adversely affect both the crystallization process and the crystal quality by inducing deviations from the ideal diffusion controlled growth and movements of crystals. The forces derive from random accelerations, called g-jitters, produced by mechanical vibrations caused by operation of equipment. Furthermore, experimentation in space has restricted access and is extremely expensive and difficult to control.

### 2.8. Magnetic Field

Both a uniform magnetic field and a magnetic field gradient can be used for protein crystal formation experiments [54]. Inhomogeneous magnetic fields are responsible for reducing the gravity that a solution feels through the action of a magnetization force and for damping of convection in the solution [55,56]. In order to suppress convection, the buoyancy forces caused by differences in mass density have to be opposed by magnetic buoyancy forces due to differences in magnetic susceptibility. When a homogeneous magnetic field is applied, an increase of viscosity is observed near the growing crystal. This effect determines a reduction of natural convection inside the crystallization solution and a decrease of the diffusion coefficient of the protein. Low gravity environments obtained by the above methods represent the better way to simulate on-Earth the reduced gravity conditions that are experienced in the space. Furthermore, an orientation effect is observed on crystals formed under high magnetic fields [52,57], which sometimes induces a change of the habit of protein crystals. Indeed, magnetic fields orient structures of proteins so that α-helix and β-sheet structures become parallel to the magnetic field direction [58]. All these phenomena seem to improve the resulting crystal quality, although a more extensive study is necessary to better understand the mechanism.

### 2.9. Electric Field

The application of external electric fields of high voltages (1–10 kV) to the protein crystallization solution has shown that it is possible to reduce crystal nucleation, and to control the kinetics of the crystallization process [59]. The electric field tends to localize the nucleation near one electrode, depending on the polarity of the protein fixed by the working pH, to decrease the nucleation time and to improve the dimensions and sometimes the diffraction quality of crystals. The electric field can create up to a three-fold increase in local protein concentration, yielding larger, sometimes better quality, crystals. Both Direct Current (DC) and Alternative Current (AC) electric fields can be utilized. The effect of an external electric field on the nucleation rate is correlated to the difference in the electrical permittivity between the liquid and the solid phase. In the low frequency region the electrical permittivity of protein crystals is larger than that of the protein solution, whereas the opposite occurs in the high frequency region, thus it is possible to reverse the electrical permittivity between the liquid and the solid by changing frequency. The effect of the external electric field on the nucleation rate increases with an increase in the concentration of the precipitant: a larger ionic strength of the precipitant corresponds to a greater effect of the external electric field on the crystallization process [60]. Comparison of the results of crystallization trials performed with the same precipitant but with different frequencies indicates that the nucleation rate can be controlled by imposing an external electric field with an appropriate frequency. The nucleation rate of hen egg white lysozyme was successfully controlled, in terms of both increase and decrease in nucleation, by application of an AC electric field [61]. A number of devices have been developed to set-up experiments under electric field [62–68]. Electric field-induced crystallization experiments can be also performed by putting the electrodes directly in contact with the protein solution (called internal EF) [69,70]. Since electrochemical reactions could occur on the electrodes, this technique needs precise control in applying the adequate current or potential. The main features of the internal EF protein crystallization, that are fast crystallization and good quality of the crystals, are associated with a higher flux of mass from the bulk of the cell to the region near one of the electrodes, where the crystallization is being favored. However, the details of the processes that cause crystallization in an electric field remain unclear, so most successes using this technique are the result of a trial and error procedure. To date these studies have only dealt with model proteins, but the remarkable results on nucleation control, size distribution and diffraction quality of crystals make the use of electric field for crystal growth very promising.

### 2.10. Stirring

Stirring of crystallization solution has been found to affect crystal nucleation and growth. Stirring enables the control of crystal growth by minimizing concentration gradient across the crystal surface. It prevents excess spontaneous nucleation and accelerates the growth of protein crystals, thus resulting in the growth of large and high-quality crystals and, in some cases, also in the production of crystals of molecules that do not easily crystallize using conventional techniques. It has been supposed that stirring increases the interactions between protein molecules, which might associate leading to the development of large clusters that might evolve into stable critical nuclei [71]. However, it is necessary to stir gently the sample solution, to avoid problems such as spontaneous nucleation, protein denaturation, and damaged crystals [72]. It is important to remember that crystal growth and crystal morphologies in a stirred solution are strongly dependent on the stirring speed [73]. Several experimental set-ups are used to apply the desired mild stirring to crystallization solutions. In particular, it is worth to mention two simple methods: the micro-stirring and the micro-floating and stirring (micro-FAST) techniques ([72] and references therein). In the micro-stirring technique the crystallization mixture is gently rotated by using a common rotating or vibrating shaker. This procedure enables an easy mixing of the solution and a control of the forced flow conditions by changing the rotation speed and type of shaker. In a micro-FAST experiment the protein solution is added to an insoluble and highly dense liquid, from which it separates: protein solution floats on top of this liquid, forming an interface. The protein crystals that grew at the interface did not contact the vessel, resulting in regular crystals with improved crystallinity. The liquid of the lower layer is mixed using a magnetic stirrer.

Several examples of successful application of stirring methods are reported in the literature: the crystal quality of human triosephosphate isomerase [74], adenosine deaminase [71], Src homology 2 domain-containing protein tyrosine phosphatase substrate-1 [75] was dramatically improved in this way.

## 3. Conventional Crystallization Methods

**Figure 3:**
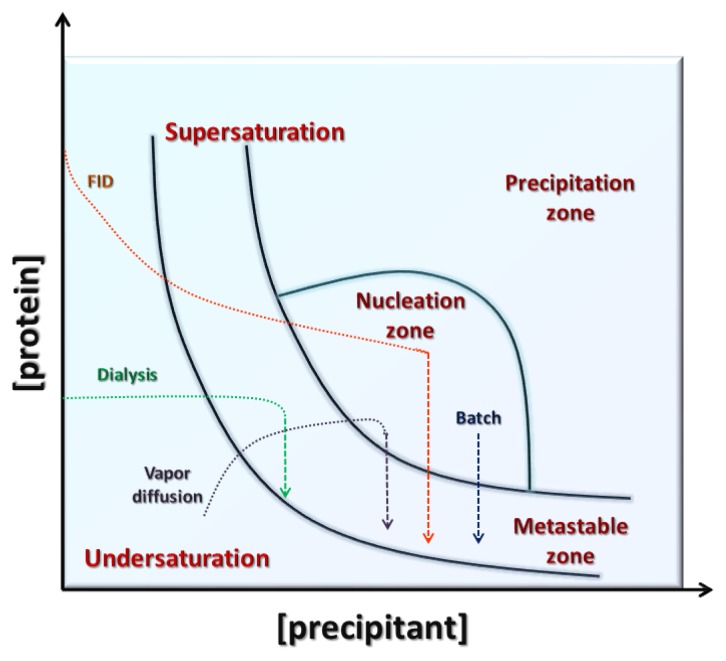
A simplified protein crystallization phase diagram. The different routes of reaching nucleation and metastable zones for the four main crystallization techniques are also shown.

A number of techniques have been developed for bringing a protein solution into a supersaturated state [9,10,14,15,36]. This paragraph is a brief summary of the conventional methods that are commonly employed in the crystallization of macromolecules: vapor diffusion, free interface diffusion (FID), batch and dialysis. A schematic illustration of a protein crystallization phase diagram is reported in Figure 3. The main crystallization methods are indicated, showing the different paths they use to reach the nucleation and metastable zones.

### 3.1. Vapor Diffusion

**Figure 4:**
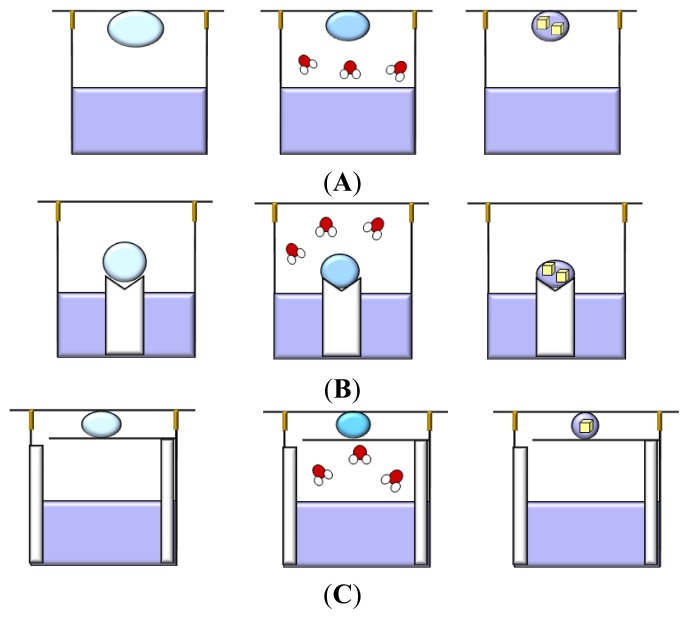
Vapor-diffusion method. A drop containing unsaturated precipitant and protein solution is placed in a well containing a reservoir with precipitant in higher concentrations. The well is hermetically sealed to prevent droplet evaporation and to allow vapor equilibration of the droplet and the reservoir. Equilibration of water vapor from the protein-containing droplet to the reservoir solution causes the protein solution to reach a supersaturation level, where nucleation and initial growth occur. (**A**) Hanging drop technique. The droplet is placed on a siliconized glass that is used to close the well; (**B**) Sitting drop technique. The droplet is placed on a small bridge inside the well; (**C**) Sandwich drop technique. The droplet is placed between two cover slips, one of which closes the well.

This technique utilizes evaporation and diffusion of water (and other volatile species) between a small droplet (0.5–10 μL), containing protein, buffer and precipitant, and a reservoir (well), containing a solution with similar buffer and precipitant, but at higher concentrations with respect to the droplet [76–78]. The wells are sealed by creating an interface of vacuum grease between the rim of each well and the cover slip, or by using, in specific cases, a sealing tape. The droplet is equilibrated over the well solution as either a hanging, a sitting or a sandwich drop (Figure 4) to allow a slow increase of both the protein and precipitant concentration that could cause supersaturation and crystal growth. In the hanging method (Figure 4A) the drop is placed on the underside of a siliconized glass cover slide, while in the sitting method (Figure 4B) the drop is placed on a plastic or glass support above the surface of the reservoir. Finally in the sandwich drop (Figure 4C) the protein mixed with the precipitant is placed between two cover slips, one of which closes the well.

The difference between the concentration of the precipitant in the drop and in the well solution causes the evaporation of water from the drop until the concentration of the precipitant equals that of the well solution. Since the volume of the well solution is much larger (500–1000 μL) than the volume of the drop (few microliters), its dilution by the water vapor leaving the droplet is negligible.

Sitting-drops are simple to prepare by hand and by robots, but sometimes crystals can adhere to the support surface, making their removal difficult. On the other hand, hanging drops are smaller in volume and more challenging to set up. A simple protocol to convert sitting-drop vapor-diffusion plating into a hanging drop vapor-diffusion experiment uses agarose gel to solidify the reservoir solution of sitting-drop trials, thus allowing the incubation of the sitting-drop plate upside down [79]. The sandwich drop method has the advantage of reducing the exposed surface area of the drop thus decreasing the rate at which the precipitant reservoir solution draws water from the droplet and slowing down the equilibration process.

Vapor diffusion is the optimal technique either to screen a large number of conditions, by varying the composition of each well solution, or to increase or decrease the concentration of the protein in the equilibrated state relatively to its initial concentration, by varying the volume of the protein mixed with the well solution when the drop is initially setup. In a recent study, the effects of diluting either protein or crystallization agents in the droplets on reproducibility and screening efficiency of protein crystallization was investigated [80]. The results indicate that reducing the initial concentration of the crystallization agent in the droplet leaving the protein concentration unchanged increases the rate of vapor diffusion and produces higher success rate of protein crystallization. In contrast, dilution of the protein while the initial concentration of the crystallization agent remains unchanged produces effects that depend on the kind of protein. These findings seem to be in contrast with those reported by Chayen *et al*., who found that slowing down the equilibration rate, by varying the distance between the reservoir and the crystallization drop or by inserting an oil barrier over the reservoir, improves the crystals size and quality [81,82]. These data clearly confirm the complexity of understanding and controlling the various factors intervening in the protein crystallization process.

### 3.2. Batch

**Figure 5:**
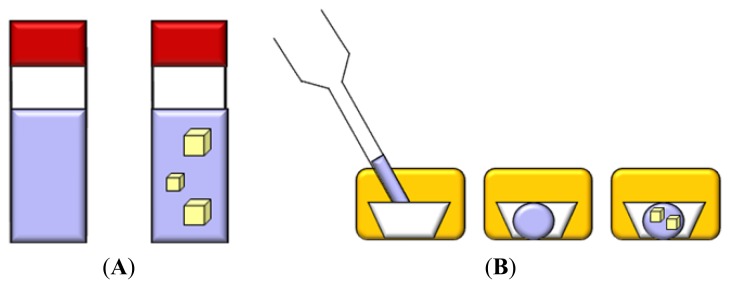
Batch and microbatch methods. (**A**) Batch method. Small vials containing pre-mixed protein and precipitant solutions at supersaturated conditions are sealed and left undisturbed; (**B**) Microbatch method. A small droplet containing both protein and precipitant at supersaturation conditions is immersed in an inert oil that prevents sample evaporation.

In the batch method the supersaturation is achieved directly rather than by diffusion. The sample is mixed with the precipitant and appropriate additives to create a homogeneous crystallization medium, then the mixture is left undisturbed (Figure 5A).

The technique can be miniaturized by immersing crystallization solution droplets as small as 0.5–1 μL into an inert oil, which prevents evaporation of the sample. This is the so called “microbatch” method (Figure 5B) [83].

In a batch experiment there is no exploration of the phase diagram, because the initial conditions remain stable for two, three weeks. To obtain well diffracting crystals, specific conditions must be fulfilled: nucleation has to start during the preparation of the drop, when relatively high concentrations of protein and precipitating solution come into contact with each other, and after mixing the solution must be in the metastable zone that guarantees an ordered growth. Although the evaporation of the water from the drop covered by oil is negligible, it does occur, and therefore over time clear droplets decrease their volume resulting in an increase in the concentrations of both protein and precipitant, which can yield precipitate or protein crystals. The main disadvantage of this method is that in many cases equilibration occurs very rapidly, thus affecting the rate of crystal growth and consequently the quality of crystals. Furthermore, alcohols, detergents, and lipids can diffuse into the oil. To avoid drying out of the droplets over prolonged periods it is possible to control the rate of decrease in volume of the droplets by regulating the vapor pressure within the crystallization tray [84]. On the other hand, varying ratios of water permeable oils to control water diffusion into the environment, it is possible to obtain more rapid crystallization [84–86].

Very recently, a new approach to perform microbatch crystallization has been proposed, the without-oil microbatch method [87]. This method is based on the idea that is possible to adapt the crystal growth conditions found with vapor diffusion experiments to the batch technique without changing the device and preserving almost the same composition of the reservoir solution. Protein and precipitant are mixed to their final concentration at the beginning of the experiment. The drop is then stored in the presence of a reservoir with the same precipitant concentration, to avoid drop evaporation. Crystallization usually occurs using 60%–80% of the concentration of the precipitant required in the hanging drop experiment. The advantage of this procedure is eliminating the most significant drawback of the under-oil microbatch method, *i.e.*, the slow evaporation of water from the crystallization drops that sometime results in the formation of salt deposits that interfere with protein crystal growth.

### 3.3. Dialysis

**Figure 6:**
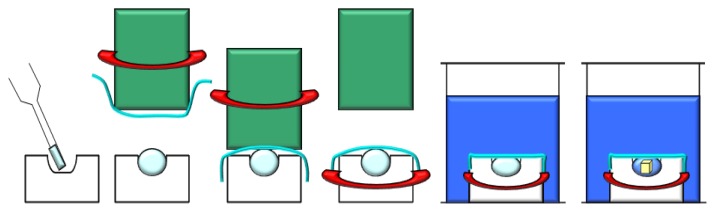
Microdialysis method. The droplet of protein solution is placed in a small button, which is covered with a dialysis membrane and closed with a thin elastic band. The button is immersed in the precipitant solution and the equilibration of precipitant molecules occurs through the membrane.

This technique utilizes diffusion and equilibration of precipitant molecules through a semi-permeable membrane as a means of slowly approaching the concentration at which the macromolecule crystallizes. Provided that the precipitant is a small molecule like a salt or an alcohol, it can easily penetrate the dialysis membrane, and the protein is slowly brought into equilibrium with the precipitant solution. Dialysis tubes can be used by itself in the case of large amounts of protein being available. Microdialysis buttons, also known as Cambridge buttons [88], offer a convenient way to perform crystallization trials with a small amount of sample. In the last case, the protein sample is placed inside a small chamber on top of the button, which is able to accommodate volumes ranging from 5 to 100 μL; the sample is covered with a dialysis membrane of appropriate molecular weight cut-off and then is placed in a reservoir containing the precipitant solution (Figure 6).

Dialysis has several advantages, including the possibility to change the reservoir composition accurately any number of times simply by moving the button from one condition to another. This allows to continuously recycle the sample solution until the correct conditions for crystallization are found. Furthermore, the rate of equilibration can be modulated by varying the differential between concentration inside and outside the membrane. On the other hand, this method does not work with concentrated PEG solutions, as they tend to draw all the water out of the button, thus resulting in protein precipitation, and does not allow to change protein concentration.

The dialysis equilibration kinetics depend on the molecular weight cut-off of the membrane, the precipitant, the concentration of components inside and outside the membrane, the dimensions of button, the reservoir volume, the viscosity of the solution, and on variation of chemical physics parameters. The rate of equilibration can be reduced to provide enhanced control over the crystallization process by a double dialysis: the button is placed inside a reservoir, which contains a certain concentration of crystallizing agent and is sealed with a dialysis membrane, which is in turn placed inside a second reservoir with a higher concentration of crystallizing agent [89].

### 3.4. Free Interface Diffusion

**Figure 7:**
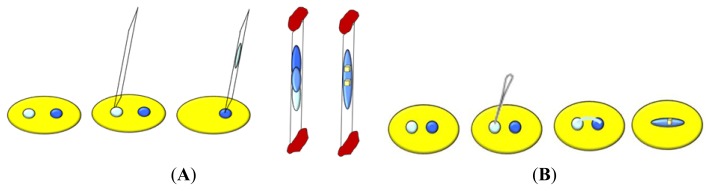
Free interface diffusion method. (**A**) Protein and precipitant solutions are placed in contact to each other in a thin capillary that is then sealed with wax. At the interface between the two solutions diffusive mixing creates a region of high supersaturation where nucleation is favored. As the protein and precipitant solutions mix, the bulk solution remains sufficiently supersaturated to support crystal growth and prevent crystal dissolution; (**B**) FID–liquid bridge method. The two drops containing the protein and the precipitant solutions, respectively, are connected by a thin liquid bridge obtained with a needle. (**A**) (**B**)

This technique relies on carefully layering the precipitant solution on top of the concentrated protein solution in a capillary, whose ends are then sealed with wax [90]. The narrow diameter of the capillary minimizes mixing from natural convection in the system. Thus, the precipitant and the protein slowly inter-diffuse and the system reaches the equilibrium by a phenomenon called counter-diffusion (Figure 7A).

When the solutions initially come into contact and diffusive mixing occurs, the region of the protein solution in the neighborhood of the interface becomes supersaturated and the ideal conditions for nuclei formation are created. As time proceeds, the two solutions inter-diffuse along the axis of the capillary and dilute each other, thus promoting the dissolution of the smaller nuclei and the growth of the larger ones. The achievement, by the free liquid diffusion (FID), of transient nucleation conditions in most cases allows to obtain high quality crystals. Thus FID can be view as a rational crystallization approach to minimize supersaturation and impurity levels at the crystal growth front and to ensure steadiness of both values. However, this method is not widely utilized by crystal growers due to the large sample volume that is needed, to the difficulty to set up a good interface, and to the presence of convective currents associated to density differences in the two solutions that disturbs the interface. The last problem can be partially solved by putting the more dense solution on the bottom or by freezing one of the two solutions [91,92]. Usually, the capillary is filled with the precipitant solution and placed in a freezer at 193 K; once the solution is completely frozen, the capillary is moved in a bed of dry ice for the addition of protein sample. At this time the system is left to equilibrate at the desired temperature. As it is demonstrated in various papers [51,93], a better way to solve the problems of the free diffusion method is to perform the experiments in microgravity, where gravity induced convection is eliminated. Currently, the nanoliter scale FID is considered particularly attractive and efficient for crystallization. It is performed by using microfluidic devices [94] that allow an ultra-small volume screening of protein crystallization conditions. Once chemical conditions conducive to crystallization have been identified, they may be exported to traditional FID by subsequent refinement. In some cases, diffraction-quality crystals have been also grown and harvested from such nanoliter-volume trials.

A variant of the FID method is the liquid bridge [95], in which a drop of protein sample and a drop of precipitant solution are placed in close proximity on a cover glass and then connected by a thin liquid bridge, obtained with the help of a small needle (Figure 7B). The liquid diffusion between the two droplets, sealed from air, may induce crystal growth.

### 3.5. Gel Crystallization

In the course of crystallogenesis investigations good results have been obtained by using gelified media [96,97]. This technique is currently utilized to lower the percentage of growth defects and to control the nucleation effectively, as in gel media the gravity-induced convection is suppressed [98]. The nuclei are trapped in the pores of gel matrix and as a consequence crystals cannot move or sedimentate [99–101]. Such a stable growth environment may favor high internal order and generate crystals with low mosaicity, with a reduced incorporation of impurities and homogeneously grown in the three dimensions. The supersaturation in a gel bead can be dynamically controlled by changing the precipitant and/or the protein concentration in the bulk solution. Manipulation of the supersaturation allows the nucleation rate to be varied and eventually leads to the production of large crystals, which are more homogeneously distributed in the gel bead. Hydrogels also provide an efficient protection of crystalline samples during handling and transport, without affecting their diffraction quality. In addition, hydrogels are completely transparent to X-rays and their presence can be crucial for preserving the diffraction properties during cryo-cooling, as was shown for instance in the case of aspartyl-tRNA synthetase crystals [102]. In many cases crystals grown in gels show a higher diffraction limit compared with crystals grown in solution [101,103], but this benefit is largely underexploited by protein crystal growers because the procedures are complicated and require large quantities (>10 μL) of sample [104]. For these reasons, in the past few years the use of gels has greatly improved by miniaturization, automation [105] and development of new approaches [106,107]. Among the typical gel precursors used for protein crystallization, such as agarose, silica, acrylamide, or sephadex, agarose gels are the most widely used hydrogels [108]. This is because agarose is stable over a wide pH range (3.0–9.0), does not release any byproducts during solidification and has a low gelling temperature (~28 °C) that makes possible its use also in the presence of heat-sensitive macromolecules. Furthermore, agarose gels show high mechanical resistance as well as elasticity even at low agarose concentrations (<6% *w*/*v*), which provides mechanical protection [105].

### 3.6. Counter-Diffusion in Gel

**Figure 8:**
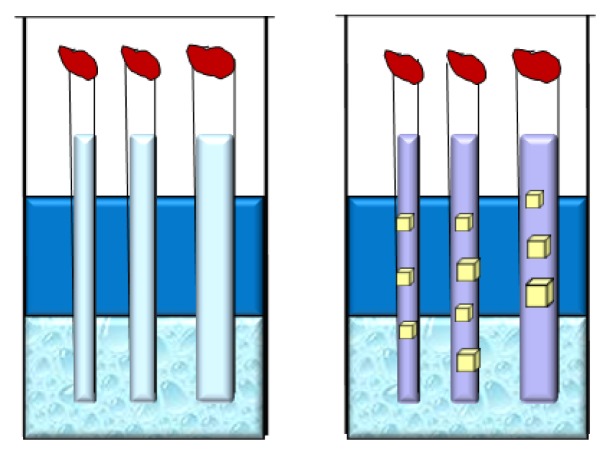
Counter diffusion–acupuncture method. Capillaries of different diameters filled with protein solution are placed in a gelled matrix, which is then overlayered with the precipitating solution. With time, diffusion of precipitant solution through the gel to the protein solution occurs.

A powerful crystallization approach has been introduced in the 90’s by Garcia-Ruiz and co-workers, who proposed to use gelled media to perform counter-diffusion experiments [109]. In practice, a gel plug is used to separate protein and precipitant solutions, otherwise one of the two solutions can be gelled before it is introduced in a capillary [110]. A subsequent improvement of this procedure is the gel acupuncture method [111] that utilizes a small crystallization vessel containing a gelified medium (silica, agarose, ...). A capillary tube is filled with the protein solution and one of its ends is sealed with wax or clay, while the other one is fixed in the gel at a penetration length of 6–7 mm. Then, the gelled matrix is over-layered with the precipitating solution. Finally, the growth box containing one or more capillary tubes is kept in a closed environment at constant temperature [112] (Figure 8).

**Figure 9:**
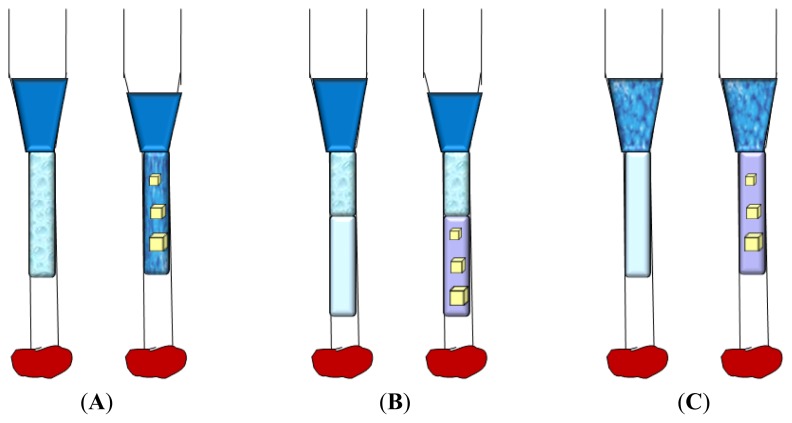
Counter diffusion techniques. In all the cases diffusion of the precipitant agent into the protein solution occurs through a gel. (**A**) The protein solution is gelified and the precipitant solution is overlayered on it; (**B**) Protein and precipitant solutions are separated by a small volume of gelled matrix; (**C**) The precipitant solution is loaded in a gelled matrix that is in contact with the protein solution.

The volumes of the gel and the precipitant agent are variables that can be selected by the operator. After the progression of the precipitating agent into the capillary, a quick increment of high supersaturation is observed and amorphous precipitation or microcrystals can be formed. Then, as the value of supersaturation decreases, fewer crystals of bigger size and quality are obtained. The gradient ensures that in different points of the capillary crystals grow under different supersaturation values. This implies that a single experiment is equivalent to many experiments with traditional methods. An important variable is the dimension of the support, as the diffusion process strictly depends on the capillary thickness. Alternatively, the gelling solution is directly added to the macromolecule stock solution and the mixture is sucked into the capillary. When the macromolecule sample is gelified, the concentrated crystallizing agent solution is poured on the top of it or directly (Figure 9A) or separated by a small volume of gelled matrix (Figure 9B). Otherwise, the capillary lower end is soaked into the solution of precipitating agent contained in a small vessel, in which the lower end of the capillary is introduced (Figure 9C).

The use of capillaries is really advantageous as it has been proven that crystals can be frozen into them for cryo-crystallography studies after diffusion of an appropriate cryo-protectant [113]. The use of counter-diffusion crystallization is facilitated by a specific crystallization device, the Granada Crystal Box (GCB) [114,115], a small and narrow box containing a capillary holder, accommodating capillaries of diameter ranging from 0.1 to 1.5 mm, and closed by a cover lid. To allow a high optical quality microscopic observation of the experiments, the GCB building material is polystyrene. The GCB has now been validated as a passive, inexpensive, and high-density crystallization apparatus for growing protein crystals in microgravity [116,117]. This device has been and is currently successfully used in several European Space Agency (ESA) and Japan Aerospace Exploration Agency (JAXA) GCF investigations on the International Space Station.

## 4. Search of Crystallization Conditions

The production of diffraction-quality protein crystals is an iterative process that can be divided into two main phases: coarse screening to identify initial crystallization conditions, followed by optimization of these conditions. Each stage is carried out with a largely empirical approach. There are a huge number of variables that can be explored in a search for crystallization conditions [118]. At the start of the crystallization of a new protein, one faces the choice of where to begin the trial and error process. Until recently, the search for crystallization conditions was initially based upon biochemical properties learned during the purification and the general biochemical characterization of that macromolecule [36]. One of the earliest screening methods, sometimes called the grid screen, employs a sampling protocol in which 24 closely related experiments are performed at a time, by varying only two factors [119]. If the results are negative the procedure continues with a second choice of factors and so on until success is achieved.

The most popular approach is based on the use of sparse-matrix screens [120]. Such screens contain a number of formulations for initial crystallization trials based on conditions that were known to have had success in protein crystal growth. Specifically, crystallization conditions that produced diffraction quality crystals are searched in the literature and the subset which samples the widest range of buffers and precipitants is identified [120]. The idea is to provide a broad enough sampling of parameter space by random combination of conditions to yield initial crystals, which may be improved later. There are many useful kits for crystallization screening available in the market today. Their use is simple, convenient and fast, but they are discontinuous in many parameters and can miss useful conditions. This problem has been partly solved by the construction of automated systems (Robots) able to rapidly perform a large number of crystallization trials and to dispense nanoliter droplets. These Robots reduce the amount of protein required for crystallization screening. Moreover, robotic crystallization trials allow for less error, enable more systematic and routine experimentation, give the possibility to collect extensive data, which can be analyzed to rationalize the approach. Finally, crystallization trials using sub-microliter drops reduce costs, make possible the exploration of a larger crystallization parameter space, shorten time of crystal formation, thus allowing a more rapid analysis of results.

An additional method is the incomplete factorial approach, a procedure that, chosen the variables to be changed, defines how to sample the variables with a minimum number of experiments [121]. Using statistical methods to analyze the results, it is possible to identify variables that are correlated, and in later stages to concentrate on their variation to optimize crystallization conditions.

The task of screen is to identify a starting point for optimization of the crystallization conditions that is usually made by a systematic approach [122]. Both screening and optimization can be performed by batch, vapor diffusion, dialysis or counter-diffusion techniques.

**Figure 10:**
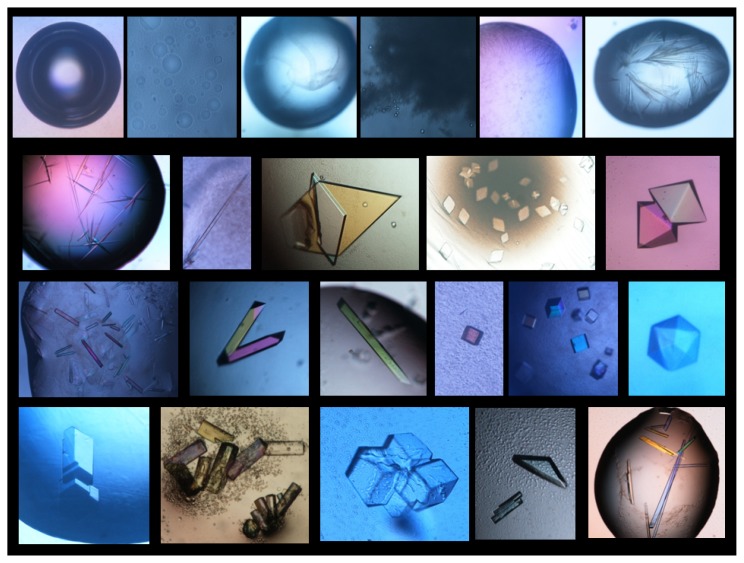
A few examples of crystallization droplets. On the first row, from left to right, limpid droplet, oil, light precipitate, heavy precipitate, quasi-crystals, brushes of needle crystals. On the last three rows well-formed crystals of different shapes.

A fundamental step in the search of crystallization conditions is the inspection and scoring of the experiments. Each trial might: (1) be clear, (2) contain amorphous/gelatinous precipitate, (3) present an heavy precipitate, (4) have oily structures and (5) contain crystal-like material or well-formed crystals, that is the best result (Figure 10).

These observations are necessary to define the variables that affect the solubility of the protein under study, to identify the more promising crystallization conditions and to determine a rational optimization strategy.

## 5. Approaches to Induce Nucleation

**Figure 11:**
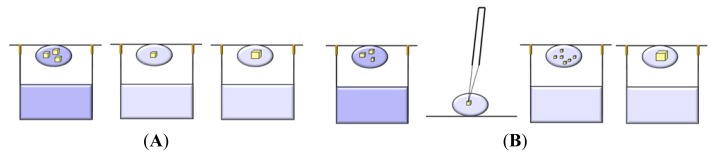
Seeding methods. (**A**) Macroseeding method. One crystal is transferred from a solution in which nucleation and initial growth occurred to a less supersaturated solution to slowly continue the growth; (**B**) Microseeding method. Crystals are fished from a supersaturated solution where nucleation and initial growth occurred, then they are crushed and the small seeds are transferred to a less supersaturated growth solution.

In many crystallization experiments the high levels of saturation needed for nucleation are not reached. An alternative mechanism is to achieve nucleation by introducing in the crystallization trial a solid material, which is termed the nucleating agent, nucleant or seed [14]. Nucleation occurs on the surface of this material, which creates a higher local concentration of macromolecules, lowers the energy barrier for nucleation and bypasses the high kinetic barrier of spontaneous nucleation. A lower level of supersaturation is required under such circumstances as the nucleation step has been bypassed. Currently, various seeding procedures are utilized: homologous seeding uses proteic nucleants, which can be auto-, when the seeds belong to the same macromolecule under study, or cross-, when the seeds belong to a macromolecule of the same family, whereas heterologous seeding uses nucleants unrelated to the target protein [123]. Furthermore, seeding techniques can be classified into three categories in dependence of the size of the seeds added to the pre-equilibrated protein solution, which was prepared on the basis of the solubility data obtained with the previous crystallization experiments. In macroscopic seeding (macroseeding) [10,14,36] one crystal, typically 5–50 μm in size, is transferred from a solution in which nucleation and initial growth have occurred to a less supersaturated solution to slowly continue the growth (Figure 11A). Prior to transfer, the crystal is usually placed in an unsaturated solution to etch its surface. This removes misoriented macromolecules or other molecules that may have poisoned the crystal surface. The microseeding method [10,14,36,124] involves the transfer of very small nuclei to solutions with metastable supersaturation (Figure 11B).

Nucleant solutions can be prepared by the crushing of crystals, the elimination of large seeds through centrifugation and the successive dilution in a stabilizing solution. Small volumes of the diluted seeds are then added to less saturated crystallization trials. Another procedure to obtain microseeding makes use of vapor diffusion to equilibrate a drop against a high concentration reservoir that is able to induce nucleation, just for the time required to start the formation of stable nuclei. Then the drop is moved in a well containing a lower concentration of precipitant agent for crystal growth. The third method is the streak seeding, which is similar to microseeding, but uses a fine hair (or animal whisker) to transport small protein crystal fragments [123]. This form of seeding is very easy and can be used to seed a series of drops rapidly. The fiber is used to touch either a crystal to remove a few seeds, or a microcrystalline precipitant, then the seeds are introduced into a fresh trial by making a straight line through the drop with the fiber. This procedure is also used to distinguish amorphous precipitate from microcrystalline one. Indeed, the former reproduces a precipitate, whereas the latter gives rise to small crystals. If necessary, the seeding can be also attempted by immerging the hair in drops containing spherulites or oils, which represent some kind of semiordered aggregation. Recently, devices able to perform automated streak seeding, either in microbatch [125] or in hanging drop [126], have been produced.

The heterologous or epitaxic seeding is a method that induces the crystal growth of a substance on a crystal face of a different one. The research of epitaxic nucleants was initiated by McPherson & Shlichta, who tested 50 different minerals as potential nucleants [127,128] and continuous actively up to now. Several different types of heterogeneous seeds have been tested in these years. The most obvious way to induce aggregation among protein molecules is the exploitation of electrostatic interactions: the engineering of charged nucleation-promoting surfaces, which interact with a protein having a net charge of the opposite sign, facilitates collisions among the sample molecules. These surfaces are chemically modified mica [129–131], poly-L-lysine surface [132,133], polymeric film [134], patterned silicon [135,136] and Langmuir-Blodgett protein thin films [137,138]. In the last case, a thin-film nano-template of the protein to be crystallized is deposited on a solid glass support, which is then used in vapor-diffusion experiments. In this way the protein itself acts as heterogeneous nucleant. Porous media, such as porous silicon [139–141], bioactive gel-glass [142], carbon nanotube based materials [143], Sephadex beads of various sizes, carbon powder, alumino-silicates, mesoporous molecular sieves [144] and zeolites of various mesh sizes, have been also successfully used, as the protein aggregation inside the porous is facilitated. Other efficient promoters of heterogeneous nucleation are the porous hydrophobic membranes that considerably reduce the time needed for crystal growth [137,138]. A number of different fibrous materials (rat whiskers, horse hair, fragments of human hair and dried seaweed) have provided really positive results [86,145], thanks to some of their properties that match those required to an efficient nucleant: ordered surface at molecular level, ionizable groups, lipid layers, local concentration cavities, nano and mesoscopic structure. The development of lithography techniques allowed the fabrication of a variety of silicon substrates with different surface characteristics [136,146] that seem to be interesting heterogeneous nucleant materials. Very recently, Saridakis *et al.* [147] have shown that molecularly imprinted polymers (MIPs) may be used as nucleation-inducing substrates. MIPs are prepared by copolymerizing functional and cross-linking monomers in the presence of a molecular template, thus creating micro-cavities with a three-dimensional structure complementary in both shape and chemical functionality to that of the template. In this case MIPs have been designed to specifically attract their template, the protein to be crystallized, and have resulted able to rebind the template and to make the crystal nucleation more effective. Interestingly, MIPs tailored for specific cognate proteins have given positive results with other proteins with a molecular weight of the same order of magnitude.

Many of the heterogeneous seeds tested in these years have provided well diffracting crystals with low mosaicity in conditions that in their absence do not induce nucleation at all. This have suggested the use of heterogeneous seeding also in initial screening experiments. In fact, recently heterogeneous nucleating agents have been added to a sparse matrix crystallization screen [148] and crystallization plates that are locally coated with fragments of human hair have been prepared [149].

## 6. Crystals for Neutron Crystallography

Determining the position of hydrogen atoms is essential to define protonation states of catalytically important residues, hydrogen bonding and solvation effects. In order to observe hydrogen atoms via X-ray crystallography, diffraction data beyond 1.0 Å are required. These high-resolution data can be achieved only with highly ordered crystals, which account for less than 1% of all crystallized proteins. A good alternative is Neutron Macromolecular Crystallography, which can provide accurate hydrogen atom coordinates, as well as hydrogen/deuterium exchange information in macromolecular crystals at a moderate 2 Å resolution. In the past, due to the lack of efficient instrumentation and the need for large crystals (~1 mm^3^), relatively few biological macromolecules have been studied by single-crystal neutron crystallography. Recent technical developments have provided orders-of-magnitude gains in efficiency and have stimulated an increasing interest in neutron protein crystallography [150]. However, the low flux of the available neutron beams still requires large fully or partially deuterated crystals.

A rational way to find the proper conditions to grow large and well-formed single crystals is to determine the crystallization phase diagram, which allows a punctual control of the parameters affecting the crystal growth process. In particular, the aim is to induct the nucleation at supersaturated conditions close to the solubility boundary between labile and metastable regions, by modulation of specific physical parameters.

The detailed knowledge of the phase diagram is at the basis of the temperature-controlled experiment (TCE), a new procedure to grow large crystals that combines the use of temperature control and seeding [151]. A crystallization drop in the metastable zone is seeded with small protein crystals. The seeds are maintained inside this region for as long as possible with the aid of temperature variations just after the crystal solution equilibrium is achieved. The temperature variation is repeated until crystals of suitable size for diffraction measurement are obtained.

Several other techniques have been developed in order to grow large protein crystals [152,153], including the two-liquid system, the top-seeded solution growth (TSSG) and the crystallization in the presence of the semisolid agarose gel (SSAG). In the two-liquid system procedure, the protein solution is added to an insoluble, transparent, colorless and highly dense liquid. The drop containing the crystallization solution separates from the liquid and floats on top of it. In this way the grown crystals do not contact the vessel and can be easily manipulated. This procedure can be applied to perform both microbatch and vapor diffusion experiments. To improve dimensions and quality of crystals, the protein can be gently stirred to minimize the concentration gradient across the crystal surface and/or slow-cooled [72]. In TSSG method the growth vessel contains a layer of an insoluble, transparent, colorless and highly dense liquid on which the protein/precipitant solution is stratified [152]. A seed crystal is hung by a thin crystal holder from the top of the vessel and is immersed in the mildly stirred crystallizing solution. With respect to other seeding procedures, this method prevents the formation of polycrystals, provides macrocrystals and allows a successful control of the crystal shape by changing the seed orientation. Finally, it has been shown that protein crystallization in the presence of a semi-solid agarose gel (agarose greater than 1.0% *w*/*v*) promotes nucleation and produces big crystals with high quality [154]. The combined use of a femtosecond laser technique realizes protein nucleation at lower supersaturation [155]. Focused irradiation of laser causes the formation of cavitation bubbles, which create a local high concentration of protein that stimulates nucleation. The presence of an high percentage agarose gel guarantees the spatial control of nucleation, the low diffusion of protein molecules and consequently the formation of large well-diffracting crystals with mechanical stability [106].

## 7. Nucleic Acid in Free or Liganded State

Crystallization of nucleic acid-containing systems presents additional problems to be taken in account with respect to crystallization of proteins. First of all, obtaining a sample that is homogeneous for length, sequence and three-dimensional structure is not trivial. Depending on their length, DNA and RNA molecules can be synthetized from constituent nucleotides, enzymatically generated *in vitro* by polymerase chain reaction (PCR) or produced *in vivo* by recombinant expression. Although synthetic methods give purer samples with respect to other approaches, they cannot be used for production of long chains. On the other hand, enzymatically generated nucleic acids often possess 3′ and/or 5′ heterogeneities, which are not desired [156]. Moreover, nucleic acid molecules, especially RNA, can adopt a wide variety of conformations with different associated functions. Indeed, in addition to various duplex structures, single-stranded nucleic acids can fold into a variety of structures, including hairpin, triplex, G-quadruplex, and i-motif structures [157], containing non-canonical base pairs. A proper choice of length and sequence of the oligonucleotide is the first step to obtain a stable well defined topology. A homogeneous folding can be induced by a controlled unfolding-refolding process. Annealing protocols are the most used: heating at 80/90 °C and subsequent slow cooling at room temperature is usually effective to obtain duplex structures, in particular in the case of DNA, whereas a flash cooling by transfer to ice water is supposed to promote hairpin formation [158]. Finally, urea-induced denaturation followed by refolding through gradual dialysis against buffer lacking urea is a less common method, but useful in the case of some RNAs [159].

The chemical nature of nucleic acids represents a hindrance to crystallization. The repetitive array of negatively charged phosphate groups on the surface of the molecule makes crystal packing particularly difficult [160]. For these reasons, it is common to engineer the constructs in order to favor the crystallizability of the molecule, rather than testing many different crystallization conditions [161]. The simplest way to engineer a nucleic acid molecule is defining its minimal 5′ and 3′ boundaries. Some biological interesting RNAs are functional domains embedded within larger RNAs. In this case it is possible either deleting functionally dispensable elements, or replacing them with motifs that promote intermolecular contacts or with specific protein binding sites in order to use RNA binding proteins to improve potential lattice contacts. Indeed, it has been hypothesized that introduction of a chemically differentiated surface, such as those provided by RNA tertiary interactions or by RNA-binding proteins, may facilitate the growth of well-ordered crystals [162].

Numerous commercial kits are specifically designed for crystallization of nucleic acids. Common precipitating agents are MPD and low molecular weight PEGs. Moreover, various additives have been found very effective for the growth of high quality crystals. These additives include polyamines such as spermine, spermidine or cobaltic hexamine chloride, non-volatile organics, and divalent cations such as zinc, calcium or magnesium.

### 7.1. DNA and RNA Quadruplexes as an Example of Nucleic Acid Structures

G-rich sequences able to form complex helical structures, known as G-quadruplexes, have been identified in many areas of the chromosomes including the telomere, gene promoters [163] and mini-satellite regions [164] and more recently in telomeric RNA [165]. These structures contain two or more stacked G-quartets and are stabilized by the presence of positive ions between them. Targeting of G-quadruplexes offers promising opportunities for treatment of several pathologies, in particular tumors, since such structures inhibit the activity of telomerase, an enzyme that is selectively expressed in cancer cells [166]. These results have stimulated much effort towards the search of G-quadruplex specific binding molecules able to stabilize the quadruplex fold. Design of small ligands that specifically and strongly bind G-quadruplexes requires a deep knowledge of these structures [167], as can only be obtained by X-ray crystallography. However crystallization of G-quadruplexes is particularly challenging, as several parameters need to be carefully controlled. Indeed, G-quadruplexes are able to adopt different topologies depending on the presence of cations and their nature, crowding, annealing procedures and so on. The stability of G-quadruplexes also varies widely; it depends not only on the identity of the stabilizing cation, but also on the DNA length and sequence [168,169]. A proper choice of the length and sequence of the oligonucleotide, in particular the length of intervening loops, and the strand stoichiometry and alignment, is the first step to obtain a stable well defined quadruplex topology. Secondly, a careful identification of the identity of the stabilizing cation is needed. Potassium ions are known to strongly stabilizes G-quadruplexes [170] and potassium buffers are usually to be preferred with respect to those containing sodium ions. Finally, as for other nucleic acids, a correct annealing protocol is severely requested to obtain a homogeneous G-quadruplex sample. Moreover, in the case of co-crystallization experiments it should be defined if the ligand has to be added to the nucleic acid before or after the annealing.

Commercial kits, which are specifically designed for crystallization of nucleic acids, do not work equally well for G-quadruplexes [171]. For example, the high concentrations of divalent cations may be detrimental for quadruplex crystallization. It is better to design home-made screening kits taking into account that: (1) exploration of a wide pH range is not necessary because most of quadruplexes crystallize in narrow neutral-slightly acidic conditions; (2) the precipitant agents giving good results are essentially MPD, PEGs with molecular weight ranging from 200 to 4000 Da and ammonium sulphate; (3) monovalent cations can be used as additives when their effectiveness in stabilization of the quadruplex structure is known; (4) divalent cations have to be used at very low concentrations [159]. The experimental conditions for G-quadruplex crystallization are rather limited in comparison with those of proteins, but this does not mean that the production of G-quadruplex crystals is easier. The nucleation conditions must be very finely screened and the growth step is usually really slow: in many cases crystals grow after several months.

### 7.2. Protein-Nucleic Acid Complexes

Complexes containing nucleic acids are inherently difficult to prepare and are stable only in some specific conditions. For these reasons in the case of protein-nucleic acid complexes a crystallization experiment requires a large preliminary work [172]. First of all, an accurate choice of both components of the complex is highly recommended. Concerning proteins that bind DNA or RNA molecules, they often have disordered or flexible loops that may interfere with the formation of a well-ordered crystal lattice [173]. Thus, to overcome this problem, identification of the minimal folded protein fragment able to exert its binding properties should be carried out by using, for example, limited proteolysis on the free or liganded protein. Beginning with the minimal sequence needed for binding, one can experiment sequences of increasing length. Considerations previously discussed in the case of free nucleic acids, also apply to protein-nucleic acid complexes. Moreover, the precise length and composition of the oligonucleotide must be experimentally determined for each new protein of interest [174]. As regards DNA, lengths that have been particularly successful in crystallization trials correspond to multiples of approximate integral or half integral turns of the DNA. In the case of RNA complexes, the choice of the oligonucleotide is even harder. First of all, most RNA-binding proteins have much less well-defined binding sites than DNA-binding proteins and identifying suitable RNA fragments that can reconstitute the functional complex requires considerable biochemical study. Furthermore, the intrinsic conformational flexibility of single-stranded RNA can complicate the reconstitution of complexes [175].

The complexes are usually obtained by mixing protein and nucleic acid at 1:1.2 to 1:1.5 molar ratios [176,177]. This because: (a) usually not 100% of the nucleic acid is perfectly folded, (b) estimation of either the protein or the nucleic acid component concentration is often approximate, and (c) an excess of nucleic acids does not represent a problem as they rarely crystallize as pure materials. Depending on the solubility of the protein, one can mix it with DNA or RNA either immediately at high concentrations or at low concentrations and then concentrate the sample by dialysis and/or by use of centricon. Once the complex is formed, a possible concern is whether purification is necessary. It has been found out that purity of single constituents is very important [178], but purification of their complex is rarely needed. Only in the case of a very stable complex, characterized by a *K*_d_ less than nanomolar, it could be purified by gel filtration before crystallization. It should be kept in mind that phosphate buffers are better to be avoided during protein purification and complex formation because phosphate ions could bind at recognition sites and prevent protein-substrate binding. High salt concentrations are also detrimental, since they may shield charges on the protein surface needed for DNA or RNA binding. For the same reason it was found that salts as precipitants have not generated as many crystals as PEG or MPD [179], even if important exceptions have also been reported (see as an example [180]). Ideal pH is neutral or slightly acidic, probably due to polar interactions between negatively charged DNA/RNA backbones and positively charged protein side chains.

If the crystallization trials are unsuccessful, another variable to analyze is the sequence of the “extra” DNA or RNA that flanks the central essential region, particularly bases at the ends of the nucleic acid molecule, which often stabilize crystal packing by making interactions with other oligonucleotide ends or protein molecules. For this reason, the use of sticky ends, single-stranded bases on nucleic acid ends, has been introduced for controlling the crystallization of protein-nucleic acids complexes [181,182].

Recently, many investigators have been involved in the search of the crystallization conditions of protein-quadruplex complexes. At the best of our knowledge, up to now only a dozen of protein-quadruplex complexes have been crystallized. The reported successful crystallizations of these complexes underline, once again, the importance of the steps regarding nucleic acid annealing and preparation of the complex [183–185].

## 8. Membrane Proteins

Membrane proteins are particularly difficult to handle, because they possess both a hydrophobic surface, which is in contact with the alkyl chains of lipids, and a polar surface, exposed to the aqueous phases on both sides of the membrane. For these reasons, the first membrane protein structure was determined only in 1980 [186]. These proteins can be extracted from natural sources by using detergents, even though their concentrations, with only few exceptions, are very low. As regards the recombinant expression, the efficiency of heterologous overproduction may be very low because of subtle differences between signal-recognition particles, cellular chaperones and foldases [187]. Furthermore, during purification steps a special attention has to be devoted to avoid unfolding, aggregation and precipitation [188]. Once a pure and homogenous sample has been obtained, the operator has to choose among five strategies for crystallization of membrane proteins, each of which presents advantages and disadvantages: detergent-based [188,189], antibody-fragment-mediated [190], lipidic cubic phase (LCP) [191], lipidic sponge phase (LSP) [192] and bicelle [193] crystallization. The latter three may be included in the general approach “crystallization in a bilayer environment”.

### 8.1. Detergent-Based

The oldest method, and so far the most popular and successful one, is the detergent-based crystallization [189,194]. Membrane proteins are isolated from the cell using detergents and the purified detergent-solubilized protein is crystallized using techniques and experimental settings routinely applied for crystallization of soluble proteins. This easy approach presents several drawbacks. First of all, detergent micelles can mask much of the surface area available for crystal contacts, hindering the formation of well-ordered crystals. Moreover, since detergents are not perfect mimic of natural bilayer, the membrane proteins may not be stable enough to produce high quality crystals, and, more seriously, they may also unfold [195]. To overcome these problems crystallization in bilayer environments has been introduced.

### 8.2. Lipidic Cubic Phase

The lipidic cubic phase (LCP), introduced by Landau and Rosenbusch [191], consists of a single lipid bilayer bent into a highly organized three dimensional lattice permeated by aqueous channels [196]. This bicontinuous lipidic mesophase is more ordered than a liquid but less ordered than a solid. The membrane protein is incorporated into this semi-solid cubic phase by mixing a buffer containing the purified protein with the component of LCP (usually monoolein) in a proper ratio [191]. On the basis of temperature-composition phase diagram for the monoolein/water system, the relevant cubic phase forms spontaneously at 20 °C above a hydration level of ~35% (*w*/*w*) water. Included into these matrices soluble and membrane proteins retain native conformation and enzymatic activity [197,198]. From 1996 to present this *in-meso* crystallization method has been greatly improved [199–203] and it has seen numerous successes in elucidating mechanisms of action of several microbial rhodopsins [204–208], in defining the crystal structure of the photosynthetic reaction centre from *Rhodobacter sphaeroides* [209], the high-resolution details of human G protein-coupled receptors bound to ligands [210], and of other free or liganded human proteins, such as the human A2A Adenosine Receptor Bound to an Antagonist [211], the human histamine H1 receptor complex with doxepin [212], the human M2 muscarinic acetylcholine receptor bound to an antagonist [213], *etc.* Recently, many efforts have been done to obtain an automatic high-throughput mode to perform cubic phase experiments [214–217]. The protein containing LCP is used in crystallization experiments with the advantage of obtaining crystals that are stabilized through extensive contacts in both the hydrophilic and hydrophobic protein regions [196]. Drawbacks are related to the practical difficulties of handling the highly viscous LCP [218], the need of specialized tools for all steps of crystallization, the use of lipases and special treatments to fish crystals out of the lipidic medium [219].

### 8.3. Lipidic Sponge Phase

In some cases the semisolid LCP liquefies before crystal formation, giving rise to a lipidic sponge phase (LSP). LSP may be thought as a swollen cubic phase with larger aqueous pores and lower long-range order. Thanks to its liquid state, LSP is easier to handle with respect to LCP and is suitable for traditional vapor-diffusion experiments [220].

### 8.4. Bilayered Micelles

In this method the lipidic media used for crystallization are bilayered micelles, also called bicelles, which are solubilized lipid bilayer disks, formed by the addition of an amphiphile (a detergent or a short-chain lipid) to a long-chain lipid in an aqueous solution. The long-chain phospholipid forms a central planar bilayer that is surrounded by the amphiphile chain protecting the hydrophobic edge of the bilayer [196]. In this medium, which somewhat mimics the *in vivo* membrane organization, the proteins are likely to retain their native structure and functionality. At temperatures below the transition point, bicelles are in a liquid state that allows their use in standard crystallization techniques and automated experiments.

### 8.5. Antibody-Fragment-Mediated

This is a method particularly helpful for membrane proteins with a very small hydrophilic surface [190]. The binding of Fv or Fab fragments to the target membrane protein is used to enlarge the hydrophilic portion of the protein, thereby providing additional surface area for molecular contacts and space for the detergent micelle. The choice of the antibody is critical as it must make a stable complex with the membrane protein in detergent solution and favor ordered intermolecular contacts. A screening of these membrane protein crystallization chaperones can be rapidly performed by using new procedures for the selection of antibodies [221–223].

Membrane proteins can form three different kind of crystals, named Type I, II and III.

**Figure 12:**
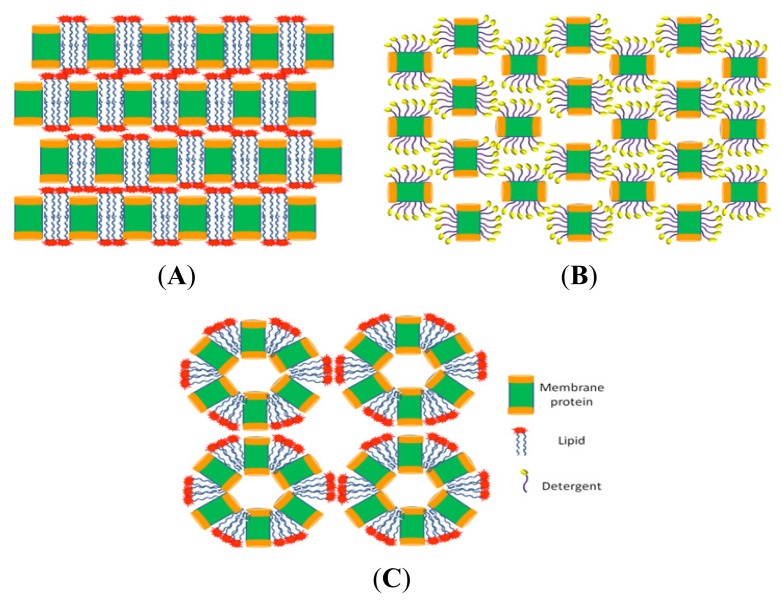
Different crystal types of membrane proteins. (**A**) Type I crystals: layers of membrane proteins stacked side by side, with hydrophobic surfaces providing crystals contacts, are stacked one on another; (**B**) Type II crystals: detergent molecules shield hydrophobic regions of membrane proteins and crystal contacts involve only polar heads; (**C**) Type III crystals: vesicular proteoliposomes forming the crystalline arrangement.

Type I crystals (Figure 12A) are formed by stacked 2D crystals or 2D layers. In each layer membrane proteins are oriented as in a bilayer, stacking side by side with hydrophobic surface providing crystals contacts. These crystals easily grow in two dimensions, but are often disordered along the third one. As expected, crystallizations in lipidic phases produce Type I crystals [224]. Detergent-based crystallization produces Type II crystals (Figure 12B), where hydrophobic regions are shielded by detergent molecules and only polar surfaces contribute to crystal packing. Usually these crystals badly diffract X-rays because they have few crystal contacts and protein molecules may be disordered [195,224]. Type III crystals (Figure 12C) are formed by vesicular proteoliposomes, which can be considered as closed spheres made by pieces of 2D membrane protein crystals. Both inner and outer surfaces of this kind of spheres are hydrophilic [225].

## 9. Real-Time Monitoring of Crystals

Structural genomics initiatives have stimulated the development of high-throughput methods for cloning, expressing and purifying proteins, setting-up crystallization trials, monitoring crystal growth, mounting crystals, and acquiring and analyzing diffraction data. A successful crystallization requires a continuous monitoring of the process and a careful scoring of the results [226]. The experimental methods more frequently used in the analysis of the crystal growth experiments and in the interpretation of their results are reported in Table 4.

One of the most critical steps is the automated observation and scoring of crystallization trials, because protein crystal morphology is large and complicated. Several approaches can be used, but unfortunately, a universal, easy strategy is still lacking. Common crystal-growth monitoring robots collect white-light macroscopic images of crystallization drops. The images can then be analysed using pattern recognition programs for the presence of crystals, microcrystals, precipitate, *etc.* However, it is extremely difficult to come up with a unique set of descriptors for crystals and a manual inspection of crystallization trials is in any case required. An alternative technique is based on the observation of crystal or microcrystal birefringence [231]. Of course, this method requires special plates and devices, since birefringence due to plastic ware can interfere. Moreover, it is known that monometric crystals, and crystals with lower symmetry in particular orientations, are not birefringent and cannot be detected by this method. A better performance is obtained by using polarization interferometry (DPI). This method allows the correlation of changes in refractive index to the thickness and density of surface layers immediately above a waveguide surface. DPI has been recently used to discriminate between the formation of crystalline and amorphous material in real time [238]. The automated setup allows the screening of a continuous or stepwise concentration gradient of a precipitating agent in each trial. In this way, DPI provides useful information for the study of nucleation and crystal growth optimization. Importantly, this technique can be used in the case of most of the crystallization methods. Another tool for routine analysis, scoring and optimization of the crystallization processes is dynamic light scattering (DLS). It has been demonstrated that the rate of the particle size growth is a good predictor of the outcome for the crystallization experiment and allows to assess the probability of obtaining macromolecule crystals before their growth starts [234]. In the evaluation of the crystallization trials it is also possible to utilize the fluorescence of tryptophan residues under UV light. However, UV-fluorescence has several drawbacks: emission of plastic ware, buffers and impurities may interfere with protein fluorescence, irradiation with UV light may damage proteins or trigger reactions, fluorescence can be quenched by various mechanisms and the protein may not contain tryptophan [251]. The deficiency in tryptophan or another fluorescent component can be effectively overcome by the addition of fluorescent probes that bind to the biomolecule under study. A more efficient screening is obtained by two-photon excited UV fluorescence; this method is characterized by insensitivity to optical scattering, which allows detection also in turbid solutions, elimination of potentially damaging out-of-plane UV excitation, and improved signal to noise ratio [252]. Finally, also Raman scattering in near-IR region represents an useful tool to identify protein crystal. In this case spontaneous fluorescence is minimal and a spatial map of the intensity of Raman bands characteristic of the protein can be produced to reveal the protein distribution, as well as to have information on secondary structures. The ideal band to be monitored is the amide I, because it is broad and intense, change very little in different proteins and does not suffer from interference of commonly used buffers, salts and precipitants. In particular, Raman confocal microscopy is ideal for monitoring crystal growth since it is non-destructive, inexpensive and rapid. It is possible to focus through transparent capillary (e.g., in FID) or directly on crystals kept in the crystallization reactor (e.g., in vapor diffusion experiments) allowing straight analysis on the sample. Indeed, no special handling is required in manipulating crystals for Raman measurements. In co-crystallization experiments two independent Raman spectra are collected on native and derivative crystals, using the same mother liquor. A Raman spectrum provides a lot of information: energy shift, intensity and polarization of the scattered light and width of the peak. Up to now, Raman microscopy proved to be a valuable support to protein crystallography in all the steps of the 3D structure determination, from the preparation of the derivative crystals [240,253] up to the interpretation of the electron density maps [254,255]. In the case of metal proteins, when resonance Raman conditions are achieved, (exciting with a wavelength within one electronic absorption band of the sample) accurate information on the coordination, spin and oxidation state of the metal can be also obtained [239].

## 10. Improvement of Crystallizability and of X-Ray Diffraction Limits

One of the most effective approach to promote crystallization uses protein engineering. In the case of proteins with highly flexible regions, such as loops or *N*- and *C*-terminal segments, these regions can be deleted after an accurate identification of the mobile region by limited protease digestion and sequence alignment of analogous proteins from different species. When the flexible fragment connects distinct domains, a useful strategy is to express and crystallize each domain independently. Other sources of sample heterogeneity are post-translational modifications, which can be eliminated or by action of enzymes that are specific for the particular chemical group or by mutation of residues that are subjected to post-translational modification. Point mutation is also applied to change surface residues that can interfere with crystal packing and/or charged residues. In this last case a net variation of protein solubility and formation of more favorable crystal contacts can be achieved. Engineering of protein surface can be also obtained by chemical modification of flexible amino acid side chains, whose mobility can hinder the formation of well-ordered crystal lattice [256]. Purified proteins can be treated with formaldehyde and dimethylamine borane complex to form a covalent bond between the amine nitrogen of lysine residues and the methyl group [257,258]. The hydrophobic nature of the dimethylated lysines reduces the solubility and in some cases favors protein-protein interactions and thus the crystallization process. A complete overview of these procedures can be found in the papers of Heras & Martin [259] and Derewenda & Vekilov [260].

Methods exist to improve the X-ray diffraction power of existing crystals. Post-crystallization methods are more crucial for large proteins and macromolecular complexes since a new, and possibly better diffracting, crystal form is usually more difficult to obtain compared to small protein targets. The most used post-crystallization methods for improvement of the diffraction properties include soaking, cross-linking, annealing [261,262] and controlled dehydration [263,264]. Soaking of protein crystals with their product analogs, inhibitors, or strong ligands can be used to stabilize protein conformation and increase the crystalline lattice regularity. Diffusion of additives, heavy metals, divalent metals into pre-grown crystals can also be tested to verify if they are able to reinforce protein-protein interface and increase the diffraction power.

The formation of intermolecular covalent bonds represents another possible way to obtain high resolution diffraction data. Cross-linkers are either homo- or hetero-bifunctional reagents able to establish inter-molecular cross-linkages. Homo-bifunctional reagents, specifically reacting with primary amine groups (*i.e.*, ɛ-amino groups of lysine residues) and soluble in aqueous solvents, such as glutaraldehyde, have been used several times to guarantee the structural rigidity of the protein. The effect depends on position and number of lysines per asymmetric unit and on the pH of harvesting solution, as the crosslinking under alkaline or acidic conditions happens by different mechanisms and gives different end products [265].

The discovery in recent years that diffraction properties of flash-cooled protein crystals may improve when they are brought to room temperature and then re-flash-cooled has had an high impact on biocrystallography [262]. Two different procedures can be used: the flash-cooled crystal is removed from the cold stream, transferred in a cryo-protectant solution for few minutes and then re-cooled [261,266] or, alternatively, the cold stream is blocked for a definite time and then restarted to re-cool the crystal. The success of this manipulation is unpredictable as it depends on the solvent content of the crystal, the cooling protocol and the solution composition. However, it should be tested, particularly when diffraction quality is low, as in many cases the static disorder of crystals is reduced during the procedure. Cryo-annealing can be considered as a fairly brutal dehydration process. In general, crystal dehydration procedures may produce very interesting results especially when the decrease of crystal solvent content is performed in a systematic and controlled manner. In the fortunate cases, this procedure induces an internal rearrangement of the crystal that results in a significantly improved quality of the diffraction data. The various techniques for dehydrating crystals and the proteins whose diffraction pattern was improved through this procedure have been summarized in a recent paper [263]. To assure the control and reproducibility of dehydration, a number of dedicated devices have been produced [267–270], some of which monitor in real time the X-ray diffraction pattern of the crystal [271–273].

## 11. Concluding Remarks

This review ends with few indications on the new tendencies in the crystallization of bio-macromolecules. A method with great potentialities is the high-pressure crystallography, which utilizes the ability of pressure to promote the crystal growth and to modify the 3D-structure [274,275]. Pressure modulation allows to explore the conformational space of the macromolecule and its dynamics, it shows conformational substates not visible with other approaches and provides very useful information for protein engineering [276]. Recently, droplet-based microfluidic systems are studied as an interesting platform to grow crystals. Microfluidics is a technology that allows users to handle small amounts of liquids inside channels of tens to hundreds of micrometers [277,278]. This enables the performance of miniaturized experiments with accurate and precise liquid transfer on scales from pico- to microliters. Microfluidic chip platforms made by X-Ray transparent material are now available [279]. Finally, a very impressive development of high-throughput pipelines for crystal growth and X-ray structure determination is in progress. The use of these robotic systems is becoming more widespread among the crystallographers; this will greatly improve the efficiency and reproducibility of experiments and will significantly reduce the time required to obtain an accurate crystallographic model of a specific bio-macromolecule.

## Figures and Tables

**Table 1:** Parameters affecting crystallization process [10,12,14,15].

Physical factors	Chemical factors	Biochemical factors
Temperature	Precipitant type	Sample purity
Pressure	Precipitant concentration	Sample homogeneity
Gravity	pH	Sequence modifications
Magnetic fields	Buffer type	Posttranslational modifications
Electric fields	Ionic strength	Chemical modifications
Dielectric properties	Sample concentration	Aggregation
Viscosity	Metal ions	Proteolysis
Vibrations and sound	Polyions	Sample pI
Time	Detergents	Ligands, co-factors, inhibitors
Equilibration rate	Heavy metals	
Nucleants	Small molecule impurities	
Methodology	Crosslinkers	
Surface of crystallization device	Reagent source	
Sample handling	Reagent formulation	

**Table 2:** Common precipitants used in macromolecular crystallization.

Salts	Organic compounds	Polymers
Ammonium sulphate	2-methyl-2,4-pentanediol	Polyethylene glycol 1000
Lithium sulphate	Isopropanol	Polyethylene glycol 1500
Ammonium acetate	Ethanol	Polyethylene glycol 2000
Sodium chloride	1,3-propanediol	Polyethylene glycol 3350
Ammonium citrate	Dioxane	Polyethylene glycol 4000
Ammonium formate	Acetone	Polyethylene glycol 6000
Sodium citrate	Butanol	Polyethylene glycol 8000
Sodium formate	Acetonitrile	Polyethylene glycol 10000
Ammonium phosphate	Dimethyl sulfoxide	Polyethylene glycol 20000
Sodium phosphate	2,5-hexanediol	Polyethylene glycol 35000
Potassium phosphate	Methanol	Polyethylene glycol monomethyl 2000
Sodium/potassium phosphate	1,3-butyrolactone	Polyethylene glycol monomethyl 5000
Ammonium nitrate	Polyethylene glycol 200	Polyvinylpyrrolidone K 15
Potassium thiocyanate	Polyethylene glycol 400	Pentaerythritolpropoxylate
Sodium/potassium tartrate	Malonic acid	Jeffamine
Ammonium tartrate	Malic acid	Polyacrylate
Magnesium sulphate	Succinic acid	Polypropylene glycol 400
Sodium nitrate	Glycerol	Modified polycarboxylates
Sodium acetate	Imidazole	
Magnesium acetate	Ethylene glycol	

**Table 3:** Classification of additives according to McPherson *et al.* [35].

Molecule	Category
Natural additives	Physiologically or biochemically relevant small molecules, such as coenzymes, substrate analogues, inhibitors, metal cofactors, or prosthetic groups
Chemical protectants	Molecules that assure protein integrity such as reductants and metal atoms scavengers
Solubilizing agents and detergents	Mild non-detergent molecules, such as sulfobetaines, low concentrations of chaotropic agents and surfactants and, in the case of membrane proteins, stronger solubilizing agents such as detergents
Poisons	Agents that partially inhibit nucleation thus facilitating the growth of few crystals of high quality, such as DMSO, DMF, low weigh alcohols and sugars
Osmolytes	Natural occurring molecules that help the protein in the adaptation to osmotic stress while maintaining native structure and function, such as TMAO, sarcosine and betaine
Non-covalent cross-linkers	Molecules able to stabilize the crystal lattice by mediating sample aggregation through reversible intermolecular interactions, electrostatic or hydrophobic, among surface groups on neighboring protein molecules
Covalent cross-linkers	Crosslinking reagents that may both reduce the conformational protein mobility and the stability of a protein-ligand complex

**Table 4:** Experimental methods used in monitoring and scoring crystallization trials.

Method	Nucleation monitoring	Control of crystallization results	Crystal defect analysis	Checking of diffraction quality
Advanced optical microscopy	[227]			
Atomic force microscopy	[228]		[229]	
Attentuated total reflectance Fourier transform infrared spectroscopy			[230]	
Birefringence		[231]		
Dynamic Light Scattering	[232–234]			
Fluorescence		[235]		
*In situ* X-ray analysis				[236]
Internal reflection fluorescent microscopy	[237]			
Polarization interferometry		[238]		
Raman		[239–241]		
Second-harmonic generation microscopy		[242]	[243]	
Small-angle neutron scattering	[244]			
Small-angle X-ray scattering	[244,245]			
Transmission electron microscopy			[246,247]	
Visible-UV		[248]		
X-ray topography				[249,250]
